# Comparison of the NIST and ENEA Air Kerma Standards

**DOI:** 10.6028/jres.103.022

**Published:** 1998-08-01

**Authors:** R. F. Laitano, P. J. Lamperti, M. P. Toni

**Affiliations:** Istituto Nazionale di Metrologia delle Radiazioni Ionizzanti, Dipartimento Ambiente, Centro Ricerche Casaccia, ENEA, c.p. 2400, Roma, Italy; National Institute of Standards and Technology, Gaithersburg, MD 20899-0001 USA; Istituto Nazional di Metrologia delle Radiazioni Ionizzanti, Dipartimento Ambiente, Centro Ricerche Casaccia, ENEA, c.p. 2400, Roma, Italy

**Keywords:** air kerma, Co-60, comparison of standards, gamma-ray standards, transfer chambers, x-ray standards

## Abstract

A comparison was made between the National Institute of Standards and Technology (NIST) and Ente per le Nuove Tecnologie l’Energia e l’Ambiente (ENEA) air kerma standards for medium energy x rays and ^60^Co gamma rays. The comparison took place at ENEA in June 1994. Two different transfer chambers from NIST were used for the comparison. The measurements were made at radiation qualities similar to those used at the Bureau International des Poids et Mesures (BIPM) (generating voltages of 100 kV, 135 kV, 180 kV and 250 kV, respectively) and with ^60^Co gamma radiation. The transfer chamber calibration factors obtained at the NIST and at the ENEA agreed with one another to 0.03 % for ^60^Co gamma radiation and between 0.1 % to 0.8 % for the medium energy x-ray beam codes.

## 1. Introduction

A comparison between the National Institute of Standards and Technology (NIST) and the Ente per le Nuove Tecnologie l’Energia e l’Ambiente (ENEA) air kerma standards was performed in June 1994. Measurements were made at the Istituto Nazionale di Metrologia delle Radiazioni Ionizzanti (INMRI) at the ENEA Research Center in Rome. The comparison was made using the medium energy x-ray beam codes adopted for comparisons by the Bureau International des Poids et Mesures (BIPM) and using ^60^Co gamma rays. Two NIST transfer ionization chambers were taken to ENEA for this comparison. These chambers were calibrated at NIST using the NIST air kerma standards before and after the measurements at ENEA. The NIST calibration factors were then compared with the calibration factors determined at ENEA.

The NIST and ENEA standards have been previously compared with the analogous standards of other national laboratories. The present measurement was the first direct comparison between the NIST and ENEA and was made to check the long term stability of the measurement equipment and to assess the effect of some modifications that occurred since previous indirect comparisons carried out by each of the two Institutions.

## 2. Irradiation Facilities

### 2.1 Radiation Beam Characteristics at ENEA

#### 2.1.1 X-Ray Beams

A 420 kV x-ray machine is used for x-ray production at ENEA. The metal-ceramic x-ray tube has a beryllium window about 2.2 mm thick and a projected focal spot size of 4.5 mm^2^. The high voltage can be varied between 50 kV and 400 kV. A potential divider is incorporated in the generator to monitor the actual voltage on the tube. With the addition of an electronic mains stabilizer, the average tube high-voltage deviations were within ± 2 % of the preset voltage.

The x-ray beam is monitored by a transmission chamber that has electrodes of polyethlene terephthalate (PTP) foils coated with aluminium and graphite so that its response as a function of energy varies by less than ± 2 % in the x-ray energy range of interest. The filters for the x-ray beam codes were mounted in a rotating wheel which was controlled remotely and turned pneumatically. The aluminum and copper filter materials used for the beam codes of this comparison contained less than ± 0.1 % impurities, and their thicknesses are known with an uncertaintity of 0.01 mm. Materials for the filters used to determine half-value layers (HVLs) had the same characteristics.

The comparison measurements were made at a distance of 100 cm and the collimator aperture was chosen to give a field size of about 10 cm diameter. The uniformity of the x-ray beam intensity, measured by means of a 0.13 cm^3^ ionization chamber, was within 0.2 % of the center out to a radius of 2.5 cm. This degree of uniformity is adequate for the comparison measurements described below. The ENEA x-ray beam codes and the other associated parameters relevant to the present measurements are given in Table 1. The mean energy values in Table 1 were experimentally determined at ENEA by means of spectra measurements [1].

#### 2.1.2. Gamma-Ray Beams

The gamma-ray beam is produced by a therapy-Type gamma unit having an activity of about 4 × 10^13^ Bq on the reference date (June 1994). The collimator of this unit was designed to minimize electron contamination. The percentage of photons scattered in the beam due to the collimator was estimated to be not greater than 3 %. The largest contribution of degraded photons originates within the source and is estimated to be about 16 % of the primary photons [2].

The chambers were positioned at a distance of 111 cm from the source, in a beam with an area of about 10 cm^2^. The beam uniformity was measured using an ionization chamber whose volume was about 0.2 cm^3^. The uniformity of the ^60^Co beam was within 0.2% of the center out to a radius of 2.5 cm. Table 2 summarizes some relevant data on the ^60^Co irradiation system used at ENEA for this comparison.

### 2.2 Radiation Beam Characteristics at NIST

#### 2.2.1 X-Ray Beams

The x-ray source at NIST is a 320 kV x-ray machine with a metal-ceramic x-ray tube. The x-ray generator is a high frequency (500 Hz), highly stabilized voltage source with an x-ray output variation of no more than ± 0.5 % in 1 h. In addition, a transmission chamber is used to normalize the output to that observed when the standard is in the x-ray beam. The x-ray tube has a window of 3 mm Be and a projected focal spot size of 5 mm^2^. The focal spot-to-chamber distance was 100 cm. At this distance, the beam diameter was 2.5 cm and the x-ray intensity was uniform to 0.1%. For the purposes of this comparison, the NIST filtrations for the beam codes listed in Table 1 were adjusted until the HVLs were as closely matched to those at ENEA as possible. The materials used for both the filtrations and HVLs were 99.99 % pure with thicknesses known to within limits of no more than ± 0.01 mm.

The monitor chamber is a transmission-type with electrodes of high purity (99.99 %) aluminum, 1.9 × 10^−3^ mm total thickness. The energy dependence of this chamber is not a factor in the manner used at NIST.

#### 2.2.2 Gamma-Ray Beams

The ^60^Co source used at NIST to calibrate the transfer chambers used for the comparisons at ENEA is a pellettized source 2 cm high × 2 cm in diameter with an initial activity 4.4 × 10^14^ Bq (reference date November 1989). At the time of the present comparison (reference date June 1994), the source activity was about 2.4 × 10^14^ Bq. The source is contained in a therapy head with a variable collimator. The calibration distance, air kerma rate, and beam size are given in Table 2. The uniformity of the photon intensity was measured with a small volume chamber (0.6 cm^3^) and was found to be within 0.1 % or less of the center within a square 2 cm on a side.

## 3. Characteristics of the Air-Kerma Standards

### 3.1 The ENEA Standards

#### 3.1.1 Free-Air Ionization Chamber

The ENEA free-air chamber for medium energy x rays is of the Attix-type and differs in geometry and mode of operation from the more conventional parallel-plate free-air chamber. One electrode of the chamber consists of two telescoping aluminium cylinders which can be independently displaced along their common axes so that the midplane of the collecting volume can remain fixed with respect to the diaphragm plane and the chamber length can be changed. An off-center aluminium rod collects substantially all the ionization produced in the chamber. Some chamber characteristics are given in Table 3. A detailed description of the chamber and its mode of operation is reported elsewhere [3, 4].

The measurement procedure is based on a subtraction method. Two ionization current readings are taken with different cylinder displacements and the difference is used to determine the x-ray air kerma or exposure (see Sec. 4). This type of chamber does not require any correction for field nonuniformity, which, in the plane-parallel free-air chambers, can constitute an important source of uncertainty [5]. On the other hand greater care in the statistical analysis of the experimental results is required for this chamber. In fact, the charge subtraction procedure can, with low signal conditions, result in greater deviations than in the case of the parallel-plate chamber.

#### 3.1.2 Cavity Ionization Chamber

The ENEA air kerma standard for the ^60^Co gamma radiation is a cavity chamber with a wall sufficiently thick to assure charged particle equilibrium for the ^60^Co gamma rays. The chamber geometry is cylindrical and both its walls and the collecting electrode are made of high purity graphite, greater than 99.985 %, with a density of 1.75 g/cm^3^ and a porosity of 14 %. A detailed description of the chamber and its mode of operation is reported elsewhere [6]. Some relevant data of the ENEA graphite cavity chamber are reported in Table 4.

### 3.2 The NIST Standards

#### 3.2.1 Free-Air Ionization Chamber

The NIST standard for x rays generated from 50 kV to 300 kV peak voltages is a parallel-plate free-air chamber, described by Wyckoff and Attix [7]. The chamber has a collector length of 10 cm and a plate separation of 20 cm. The tungsten alloy defining aperture is 1 cm in diameter and 1 cm long in the direction of the beam. The air-attenuation path is 30.8 cm. Table 5 summarizes the characteristics of the NIST x-ray standard.

#### 3.2.2 Cavity Ionization Chambers

The cavity ionization chambers used for the NIST ^60^Co gamma-ray standard were fabricated from reactor-grade high-purity graphite, following the design of Wyckoff [8]. The spherical shape was chosen in order to allow the standards to be based on a homogeneus group of six chambers of different volumes and to present a uniform, symmetrical, chamber aspect to the source. Details of the chambers, their construction, and corrections are given by Loftus and Weaver [9]. Table 6 gives the dimensions of the graphite ionization chambers.

## 4. Exposure Measurement

### 4.1 Free-Air Chambers

The exposure rate measured by a parallel-plate free-air chamber, like the NIST chamber, is given by [7]

$$\dot{X}=\frac{I}{\rho SL}\Pi_{i}k_{i},$$ (1)

where *I* is the mean value of the currents measured at positive and negative chamber polarity, respectively. In the determination of *I*, the NIST free-air chamber uses only negative polarity with rerspect to the collecting electrode. The polarity difference has been found to be negligible. All ionization currents are corrected to a relative humidity of 0 %, a pressure of 101 325 Pa (1 standard atmosphere), and a temperature of 0 °C. This is referred to as “reference conditions.” In Eq. (1) the quantity *Π_i_k_i_* includes all the correction factors required for this type of measurement [7]. The determination of the correction factors for the NIST free-air chamber was made by the procedure described in Ref. [7]. The factors related to this comparison are shown in Table 7.

The ENEA medium-energy free-air chamber differs in mode of operation from the parallel-plate free-air chamber (see Sec. 3.1.1). The exposure rate is given by [3]

$$\dot{X}=\frac{\left(I_{E}-I_{C}\right)}{\rho S\Delta L}\Pi_{i}k_{i},$$ (2)

where *I*_E_ and *I*_C_ are the values of the ionization currents measured under conditions of collapsed and extended chamber, respectively, in dry air at reference conditions; *ρ* is the density of dry air at reference conditions; *S* is the standard chamber diaphragm area; Δ*L* is the change in the free-air chamber collecting volume length from collapsed to extended chamber conditions; and the product *Π_i_k_i_* [3, 7] inlcudes all the correction factors used wit the free-air chamber measurement. The polarity effect is negligible for this type of chamber, therefore, ionization current measurements are made only at positive chamber polarity. The correction factors for the ENEA chamber, at the beam codes used for the comparison, were determined according to Ref. [4] and are shown in Table 8. At air kerma rates lower than 0.2 m Gy s^−1^ the saturation correction, *k*_sat_, was determined by extrapolation of the experimental curve (*I*^−1^, *V*^−1^) to *V*^−1^ = 0, to obtain the saturation current *I*_sat_. A correlation coefficient very close to unity was obtained for the linear plot (*I*^−1^, *V*^−1^) confirming the predominant presence of initial recombination [10]. At air kerma rates in the range from 0.2 mGy s^−1^ to 2 mGy *s*^−1^, as used for the present comparison, a straight line was instead obtained from a (*I*^−1^, *V*^−2^) plot. This corresponds to a more pronounced effect of volume recombination [10, 11].

### 4.2 Cavity Chamber

The exposure rate measured by a graphite cavity chamber can be expressed by [12]

$$\dot{X}=\frac{I}{V\rho }{\left(\frac{L}{\rho }\right)}_{\text{air}}^{C}{\left(\frac{\mu_{\text{en}}}{\rho }\right)}_{C}^{\text{air}}\Pi_{i}k_{i},$$ (3)

where *I* is the mean value of the currents measured at positive and negative chamber polarity respectively, in dry air at reference conditions; *ρ* is the density of dry air at reference conditions; *V* is the chamber collecting volume, ${\left(\frac{\mu_{\text{en}}}{\rho }\right)}_{C}^{\text{air}}$ and ${\left(\frac{L}{\rho }\right)}_{\text{air}}^{C}$ are the mass energy absorption coefficient ratio of air to that of graphite and the restricted mass stopping power ratio of graphite to that of air, respectively, and the product Π*_i_k_i_* includes all the corrections needed for exposure measurement with a cavity chamber [12, 13]. The correction factors (updated in 1986) for the NIST cavity chambers were determined according to Ref. [9] and are shown in Table 9.

The correction factors for the ENEA chamber were determined according to Ref. [5] and are reported in Table 10. At the air kerma rate used for this comparison (see Table 2), the saturation correction *k_s_*_at_ was determined by extrapolating the experimental curve (*I*^−1^, *V*^−1^) to *V*^−1^ = 0, to obtain the current *I*_sat_ in full saturation condition. Since this plot was linear it was assumed that initial recombination is predominant [10].

For the ENEA chamber the correction due to the field nonuniformity in the chamber volume was re-evaluated to improve the values previously used. This correction, traditionally described by the product of the two factors *k*_an_ and *k*_rn_ [12,13], was replaced by the product of the factors *k*_pn_ and *k*_npn_ defined by Bielajew and Rogers [14, 15]. The factor *k*_pn_ corrects for the effects due to the axial and radial non- uniformity of the field across the chamber. Only the effects due to a purely *r*^−2^ diverging field (point source) are accounted for by *k*_pn_. The factor *k*_npn_ accounts for the nonuniformity of the field due to nonpoint source effects and for the scatter from the collimators and the room. For the experimental conditions at ENEA, the factor *k*_pn_ was calculated on the basis of the Kondo and Randolph [16] and Bielajew data [14], as a function of the chamber dimensions. To determine the factor *k*_npn_, the field uniformity across the chamber in the beam transverse direction was measured. This measurement was made by 0.13 cm^3^ ionization chamber. The results showed a constant distribution, within the experimental uncertainty, up to radial distances of 1 cm (the outer chamber radius) from the beam axis. The results obtained also included the effects of the radial non-uniformity due to the beam divergence. These effects are not included in the definition of *k*_npn_ and therefore should be subtracted. On the other hand, these effects cannot be detected separately when performing a measurement of field uniformity. The correction for this latter effect was then calculated according to the chamber size and to the chamber to source distance. From this calculation, the correction was found to be negligible. Thus, taking into account the above experimental results, the factor knpn was set equal to unity.

A specific comment should be made regarding the wall attenuation and scatter correction factor *k*_wall_ [12, 13]. From the Bielajew and Rogers Monte Carlo calculations [15, 17], consistent differences were obtained for these factors with respect to the values resulting from the traditionally adopted extrapolation measurements. Such differences are of the same order for the NIST and the ENEA cavity chambers. Therefore the comparison results between the two standards would not substantially change if the new calculated values of *k*_wall_ were adopted for both the standards. However, it was decided to reconsider the possibility of changing the *k*_wall_ values only at the time when experimental confirmation of the Bielajew and Rogers theoretical results become available. To this end, an experimental research program has been initiated at ENEA.

## 5. Air Kerma Determination

The air kerma values were calculated from the exposure according to the relationship [19]

$$K_{\text{air}}=X\frac{W}{e}\frac{1}{\left(1-g\right)},$$ (4)

where *W* is the mean energy required to produce an ion pair in air; *e* is the elementary charge; and *g* is the mean fraction of the secondary electron energy that is lost to bremsstrahlung. The values used for these physical parameters are *W*/*e* = 33.97 J · *C*^−1^ [20], *g* = 3.2 × 10^−3^ for ^6O^Co gamma radiation, and *g* is in the range from 1 × 10^−4^ to 3 × 10^−4^ for x-ray beam codes used in this comparison [21].

## 6. The ENEA Charge Measuring System

**Fig. 1:**
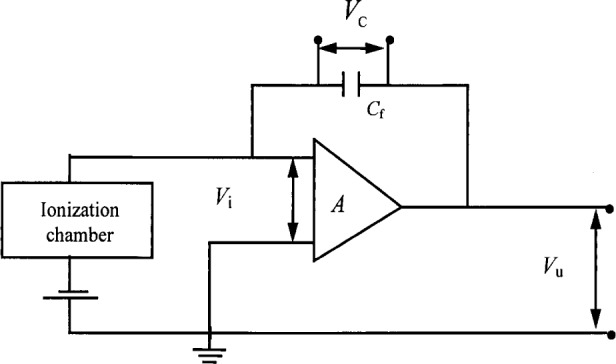
Schematic diagram of the current integrator used for charge measurement at ENEA.

The same type of charge-measuring system was used at ENEA, for the free-air chambers, the cavity chamber, the transfer chambers and the monitor chambers, respectively. This system was expressly designed for accurate measurements of the ionization currents produced by irradiation in ionization chambers of various volumes. Typically, these currents are in the range from 10^−14^ C · s^−1^ to 10^−8^ C · s^−1^ and can be determined with a relative standard (i.e., one standard deviation estimate) uncertainty from 0.1 % to 0.2 %. The charge measuring system is based on a high-gain negative-feedback MOSFET amplifier with a capacitive feedback element as shown in Fig. 1. The amplifier of the electrometer has an input impedance R > 2 × 10^14^ Ω and a gain *A* = 10^5^. For the measurement conditions described above, the potential across the capacitance chamger, *V_i_*, is forced to be near zero and constant because *V_i_* = 10^−5^ *V*_u_, where *V*_u_ is the amplifier output. Therefore, the electrical field inside the chamber and the efficiency for ion collection will be constant. According to the circuit in Fig. 1, the current throught the amplifier is negligible. The charge produced in the ionization chamber is transferred to the measuring system, as the values of the amplifier input resistance and gain are high (*R* > 2 × 10^14^ Ω and *A* = 10^5^). Under these conditions, the voltage across the feedback capacitor *C*_f_ is practically the same as the circuit output voltage, *V*_u_. Then the current through the feedback capacitor is the same as the current *I* to be measured, where

$$I=\frac{C_{f}\Delta V_{u}}{\Delta t},$$ (5)

and where Δ*V*_u_ is the change in the feedback capacitor voltage after the time interval Δ*t* and *C*_f_ is the capacitance of the feedback capacitor.

Two more features characterize the circuit shown schematically in Fig. 1. Since the change of *V*_u_ with time during chamber irradiation is linear, the slope Δ*V*_u_/Δ*t* is constant. The input terminal, being at virtual ground, is essentially isolated from the input circuit and the magnitude of the voltage on *C*_f_ does not affect the ionization current supplied from the chamber. If this were not so, the charge build-up in *C*_f_ would be exponential. The linearity of the capacitor charge during measurements is periodically checked by linear regression. Since the stray capacitances are reduced by the gain of the electrometer, it is possible to use a long signal cable. For a signal cable length of 15 m and a capacitance typically 0.1 nF · m^−1^, the total capacitance is 1.5 nF. In the described circuit, the signal cable capacitance is decreased to about 0.015 pF, as the amplifier gain is 10^5^.

The total stray capacitance due to cables, amplifier and ionization chamber was measured at ENEA and found to be less than 0.01 pF. This value is within the calibration uncertainty of the standard capacitors used. For the smaller value of the feedback capacitance, about 100 pF, the percent loss of charge trapped on the stray capacitance is about 0.01 %.

The standard capacitors used at ENEA as feedback element are high quality polystyrene capacitors with very high insulation resistance (*R* > 10^14^ Ω), low fractional temperature coefficient (− 0. 01 %/°C) and capacitance value that is very stable over a long time period. The capacitors are calibrated twice a year with a relative standard uncertainty of 0.05 % and the maximum fractional deviation observed over a period of 5 years is less than 0.1 %. Each capacitor is mounted in a aluminium box for electromagnetic shielding and physical protection. A number of polystyrene capacitors, of nominal capacity from 100 pF to 100 nF, are available for current measurements over the range of interest. The appropriate capacitor is selected according to the chamber volume and the air kerma rate. The standard capacitors are mounted in a sealed box to keep them dry. The value of the capacitor is always corrected for temperature variations during measurements and calibration.

Typical leakage currents for the ionization standard chambers used at ENEA, are less than 5 × 10^−15^ A and are subtracted from the measured currents in order to obtain the true signal. A personal computer (PC) with specially designed software was used to perform the series of measurements with the related experimental apparatus (see Figs. 2a and 2b). At the end of each series of measurements the standard capacitor is automatically discharged and after a preset time a new series of measurements is started. Computer data acquisition includes ambient temperature, pressure, and humidity data supplied by the respective probes interfaced with the computer which automatically processes the signals and calculates the appropriate corrections. The final results are presented in a printed format along with the statistical parameters. For x-ray measurements the charge measuring systems for both the standard chamber and monitor chamber are remotely controlled simultaneously.

## 7. Comparison Procedure

In the present comparison two NIST transfer chambers (NIST-T1 and NIST-T2, respectively) were used. Both the NIST transfer standards were spherical chambers constructed of air equivalent plastic. The nominal volume of the two chambers was the same, 3.6 cm^3^, with an outside diameter of 1.9 cm and a wall thickness of 0.25 mm. Equilibrium caps were used for measurements with the ^60^Co gamma beam. A collecting voltage of − 300 V was used for x rays, while a collecting potential of − 500 V was used for the ^60^Co gamma-ray measurements.

Both the NIST transfer chambers were calibrated against the NIST and the ENEA standards. The calibration factors determined at ENEA were compared with the calibration factors determined at NIST before and after the measurements at ENEA. The air kerma measurements using the ENEA standards were repeated before and after the NIST transfer chambers calibration. For each chamber, either the primary standard or the transfer chamber, a series of six groups of five charge measurements was made. The x ray calibration factors determined at NIST were based on an average of 18 calibrations. The ^60^Co calibration factors determined at NIST were based on an average of 22 calibrations for each of the NIST transfer standards. The ^60^Co calibrations at NIST were made in part before and in part after the measurements at ENEA. A summary of the NIST measurements is given in Table 11.

### 7.1 Correction Factors *k*_sat_ for the Transfer Chambers

The recombination correction factors *k*_sat_ were determined at NIST for each transfer standard for the range of air kerma rates used during the measurements at NIST and at ENEA. For the NIST-T2 chamber, the recombination correction at − 300 V is estimated to be 1.0029.

The recombination for the NIST-T1 chamber at − 300 V is represented by the equation

$$k_{\text{sat}}=1.0025+\left(0.000\;689\;{\text{mGy}}^{-1}\;s\right){\dot{K}}_{\text{air}},$$ (6)

where ${\dot{K}}_{\text{air}}$ is the air kerma rate. At the collecting potential of − 500 V used for the ^60^Co measurements at NIST and at ENEA, the recombination was found to follow the equations below for the transfer chamber NIST-T1 and NIST-T2, respectively:

$$k_{\text{sat}}=1.00132+\left(0.000\;178{\;\text{mGy}}^{-1}s\right){\dot{K}}_{\text{air}},$$ (7)

$$k_{\text{sat}}=1.00073+\left(0.000\;177{\;\text{mGy}}^{-1}s\right){\dot{K}}_{\text{air}},$$ (8)

where ${\dot{K}}_{\text{air}}$ is the air kerma rate. The correction factors *k*_sat_ for NIST transfer chambers were also determined at ENEA at the air kerma rates shown in Table 2 for ^60^Co gamma rays. The saturation current *I*_sat_ was determined by extrapolating the experimental curve (*I*^−1^, *V*^−1^) to *V*^−1^ = 0. Since this plot was linear, it was assumed that initial recombination was predominant [10]. The *k*_sat_ correction factors determined at ENEA were 1.0022 and 1.0009 for NIST-T1 and NIST-T2 chambers, respectively, as compared to 1.0017 and 1.0011, which would be predicted from Eqs. (7) and (8).

## 8. Measurement Uncertainties

The experimental uncertainties were evaluated according to the Comité International des Poids et Mesures (CIPM) recommendations [22, 23]. The combined uncertainty was obtained by summing in quadrature the uncertainties of Type A, evaluated by statistical methods, and those of Type B, evaluated by other methods. The uncertainties of Type A were evaluated as experimental standard deviations of the mean. The uncertainties of Type B were evaluated by nonstatistical methods to approximate a standard deviation.

### 8.1 Uncertainties for Measurements at ENEA

For the ionization current measurements at ENEA, the standard uncertainty *u_i_* of Type A was evaluated as the standard deviation of the mean for the series of six groups, each group consisting of five measurements. Then one has

$$u_{i}=\frac{1}{\sqrt{6}}\sqrt{\frac{1}{(6-1}\sum_{j=1}^{6}{\left({\bar{x}}_{j}-\bar{\bar{x}}\right)}^{2}},$$ (9)

where ${\bar{x}}_{j}$ is the mean of the five measurements $\left({\bar{x}}_{ij}\right)$ of the *j*th group, and $\bar{\bar{x}}$ is the mean of the mean values of the six groups.

In order to check the existence of a possible trend within each series, two independent estimates of the variance of the mean of a group were performed. Two variances, $S_{\text{st}}^{2}$ and $S_{\text{mt}}^{2}$, denoted as short-term and medium-term standard deviations, were determined. The Fisher-test was used to exclude significant differences between *S*_st_ and *S*_mt_ and to infer the statistical independence among the measurements.

The short-term relative standard deviation of the mean of a group (*S*_st_) is given by the mean of the relative standard deviations $\bar{\left(\frac{u_{x_{\text{ij}}}}{x_{j}}\right)}$ of individual readings (*x_ij_*) within each group divided by the square root of the number of readings in a group:

$$S_{st}=\frac{1}{\sqrt{5}}\bar{\left(\frac{u_{x_{ij}}}{x_{j}}\right)}=\sqrt{\frac{1}{5\times 6}\sum_{j=1}^{6}{\left(\frac{u_{ij}}{x_{j}}\right)}^{2}}.$$ (10)

The medium-term relative standard deviation of the mean of a group (*S*_mt_), is given by the relative standard deviation $\left(\frac{u_{{\bar{x}}_{j}}}{{\bar{x}}_{j}}\right)$ of the mean $\left({\bar{x}}_{j}\right)$ of each group. The mean values of the various groups are treated as if they were individual values:

$$S_{\text{mt}}=\frac{u_{x_{j}}}{\bar{x}}=\sqrt{\frac{1}{\left(6-1\right)}{\sum_{j=1}^{6}\left({\bar{x}}_{j}-\bar{\bar{x}}\right)}^{2}}.$$ (11)

Tables 12 and 13 summarize the uncertainties evaluated for the measurements with the ENEA medium-energy free-air chamber and cavity chamber respectively. The relative combined standard uncertainty of the air kerma rates determinated for medium-energy x rays using the free-air ionization chamber was 0.39 %. The relative combined standard uncertainty of the air kerma rate determinated for the gamma-ray beam using the graphite cavity ionization chamber was 0.44 %. The uncertainty of the NIST transfer chambers calibration factor determined at ENEA was evaluated in the same way (see Table 14), resulting in a relative combined standard uncertainty of less than 0.5 %, both for x- and gamma radiation.

### 8.2 Uncertainties for Measurements at NIST

The experimental uncertainties at NIST were evaluated according to Ref. [24]. For all the measurements made with the NIST transfer chambers (NIST-T1 and NIST-T2) with both the x-ray beam and ^60^Co gamma rays, the within-group average relative standard deviation was 0.01 %, with a range from 0.00 % to 0.03 %. The between-group relative standard deviations ranged from 0.03 % to 0.08 %, with an average of 0.05 %.

Tables 15 and 16 summarize the uncertainties associated with the NIST medium-energy free-air chamber and with the NIST graphite cavity chamber standards, respectively. The relative combined standard uncertainty of the air kerma rate for the x-ray beam codes, using the NIST free-air chamber, was 0.38 %. The relative combined standard uncertainty of the gamma-ray beam air kerma rate using the NIST graphite chamber standards was 0.41 %.

## 9. Results and Conclusions

The average x-ray calibration factors determined at NIST were plotted as a function of HVLs in Cu in order to account for slight differences in HVLs between NIST and ENEA. At the 100 kV beam code used at ENEA, an estimate was made for an equivalent HVL in Cu by using the ratio of Al HVLs (4.00 mm/3.896 mm). The NIST calibration factors predicted at the ENEA HVLs using these least-square fits are reported in Table 17. Table 17 also shows the percent deviations between these predicted calibration factors for the NIST transfer chambers and those determined at ENEA. The results obtained with each of the two NIST transfer chambers agree within the statistical uncertainty of the measurements. The maximum relative deviation was within the range from 0.03 % to 0.93 %, depending on the radiation quality. The percent deviations between the ENEA and NIST results do not appreciably change whether they refer to measurements with the NIST-T1 or the NIST-T2 chamber. Therefore, the mean value of these deviations was taken as the significant figure for the ENEA-NIST comparison. This is shown in Table 17 where the deviations referring to each transfer chamber are reported together with the mean deviation mentioned above. The relative mean deviations in air kerma measurements at the four x-ray beam beam codes are from 0.1 % to about 0.8 %. The relative mean deviation in air kerma measurements at the 60Co gamma-ray is less than 0.03 %.

The deviations between the ENEA and NIST air kerma standards are of the same order of magnitude as the combined uncertainty typical of these types of standards. The results of this ENEA-NIST comparison can therefore be considered satisfactory.

## Figures and Tables

**Fig. 2(a):**
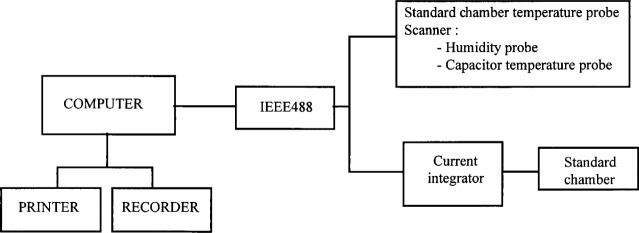
Schematic diagram of the charge measuring system at ENEA: system for measurements at ^60^Co gamma-ray with cavity chamber.

**Fig. 2(b):**
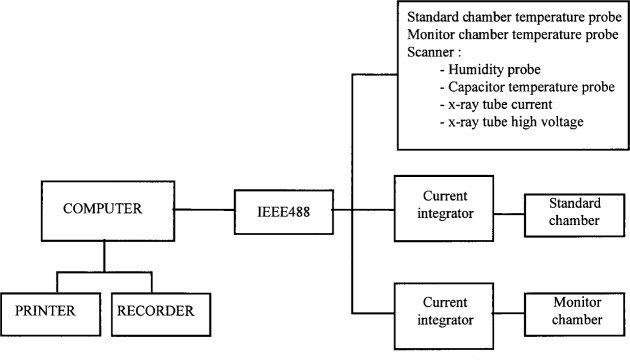
Schematic diagram of the charge measuring system at ENEA: system for measurements at x-ray machine with free-air chamber.

**Table 1:** Medium energy x-ray beam codes at NIST and at ENEA

Beam code			P6	P7		P8	P9
Mean energy		(kev)	44	68		93	139
Peak voltage		(kV)	100	135		180	250
ENEA	Inherent filtration	(Be, mm)	2.2	2.2		2.2	2.2
	Added filtration	(Al, mm)	3.49	4.08		4.06	4.02
	Added filtration	(Cu, mm)		0.17		0.43	1.48
	Half value layer	(mm)	Al, 4.00	Cu, 0.50		Cu, 1.00	Cu, 2.50
	Air kerma rate	(10^−4^ Gy s^−1^)	5.2	4.9		5.6	5.4
	Focus-reference plane distance				100 cm		
	Beam diameter in reference plane				10 cm		
NIST	Inherent filtration	(mm Be)	3	3		3	3
	Added filtration	(mm Al)	3.024	1.495		1.518	1.522
	Added filtration	(mm Cu)		0.2413		0.4936	1.5944
	Half value layer	(mm)	Cu, 0.145	Cu, 0.483		Cu, 0.967	Cu, 2.493
	Air kerma rate	(10^−3^ Gy s^−1^)	0.9; 1.8	1.7		1.8	2.2
	Focus-reference plane				100 cm		
	Beam diameter in reference plane				7.5 cm		

**Table 2:** Measurement conditions at ENEA and at NIST, for ^60^Co gamma radiation

		ENEA	NIST
Source activity^a^	(Bq)	4.2 × 10^13^	2.4 × 10^14^
Source diameter	(mm)	20	20
Source height	(mm)	20	26
Source-reference plane distance	(cm)	111	146
Beam size at the reference plane	(cm)	10	6.3
Air kerma rate^a^	(Gy s^−1^)	2.3 × 10^−3^	2.0× 10^−3^

a Approximate value at the time of measurements

**Table 3:** Principal characteristics of the ENEA medium-energy free-air ionization chamber

Change in chamber length (collapsed and extended)	(cm)	20.00
Aperture diaphragm diameter	(mm)	10.008
Aperture diaphragm thickness	(mm)	12.75
Measuring volume	(cm^3^)	15.73
Internal diameter	(cm)	30
Polarizing potential	(kV)	+ 5.0
Leakage current	(Cs^−1^)	< 1 × 10^−14^
Defining plane of aperture to chamber center distance	(cm)	40.3

**Table 4:** Relevant characteristics of the ENEA graphite cavity chamber

Diameter of cavity	(cm)	1.1
Height of cavity	(cm)	1.1
Collecting electrode diameter	(cm)	0.2
Collecting electrode height (cm)	1	
Cavity volume	(cm^3^)	1.022
Wall thickness	(cm)	0.4
Polarizing potential	(V)	± 300
Polarity effect, *I*^+^/*I*^−^		1.003
Leakage current	(A)	6×10^−15^

**Table 5:** Principal dimensions and characteristics of the NIST free-air chamber

Collecting electrode length	(cm)	10.08
Aperture diaphragm diameter	(mm)	10.00
Aperture diaphragm thickness	(mm)	10.00
Measuring volume	(cm^3^)	7.91
Plate separation	(cm)	20.0
Plate height	(cm)	26.8
Polarizing potential	(kV)	− 5.0
Leakage current	(C s^−1^)	< 5×10^−14^
Defining plane of aperture to chamber center distance	(cm)	30.8

**Table 6:** Principal dimensions and characteristics of the NIST graphite cavity chambers

Chamber code	Volume	Net volume	Outside diameter	Graphite density	Radial wall thickness	
	(cm^3^)	(cm^3^)	(cm)	(g/cm^3^)	(cm)	(g/cm^2^)
1	1.140	1.131	2.065	1.73	0.398	0.688
10	10.088	10.069	3.428	1.72	0.3755	0.647
30	30.262	30.24	4.607	1.74	0.3751	0.653
50-1	51.943	51.634	5.34	1.73	0.3652	0.632
50-2	50.425	50.089	5.58	1.73	0.5085	0.880
50-3	50.460	50.155	5.80	1.73	0.6129	1.060

**Table 7:** Correction factors for the NIST free-air ionization chamber

Beam code	Air-kerma	*k*_a_	*k*_sc_	*k*_e_	*k*_f_	*k*_sat_	*k*_d_
	rate (mGy/s)	air attenuation	scattered radiation	electron loss	aperture penetration	saturation	field distortion
P6	0.878	1.0106	0.9937	1.0000	1.0018	1.0012	1.0015
P6	1.758	1.0106	0.9937	1.0000	1.0018	1.0017	1.0015
P7	1.701	1.0072	0.9951	1.0010	1.0018	1.0017	1.0015
P8	1.783	1.0060	0.9957	1.0030	1.0018	1.0017	1.0015
P9	1.476	1.0049	0.9964	1.0050	1.0018	1.0015	1.0015
P9	2.223	1.0049	0.9964	1.0050	1.0018	1.0019	1.0015

**Table 8:** Correction factors for the ENEA medium-energy free-air ionization chamber

		Beam code		
Correction factor	P6	P7	P8	P9
Saturation, *k*_sat_^a^	1.0017	1.0020	1.0025	1.0030
Scattered radiation-electron loss, *k*_e_	0.993	0.995	0.996	0.999
Wall penetration, *k*_w_	1.000	1.000	1.000	1.000
Aperture penetration, *k*_t_	1.000	1.000	1.000	1.000
Polarity effect, *k*_±_	1.0000	1.0000	1.0000	1.0000
Air attenuation, *k*_a_	1.013	1.009	1.009	1.007

a At air kerma rate used for the present comparison specified in Table 1.

**Table 9:** ^60^Co correction factors for the NIST graphite cavity chamber, as of 1986

Chamber code	Wall absorption	Stopping-power ratio	Energy-absorption coefficient ratio	Stem scatter		Product of correction factors^a^
	(*k*_c_)	${\left(L/\rho \right)}_{\text{air}}^{C}$	${\left(\mu_{\text{en}}/\rho \right)}_{C}^{\text{air}}$	(*k_st_*)	*k*_cep_	
1	1.0168	0.9999	0.9985	0.9982	0.9950	1.0083
10	1.0216	0.9994	0.9985	0.9992	0.9950	1.0135
30	1.0220	0.9992	0.9985	0.9992	0.9950	1.0137
50-1	1.0227	0.9991	0.9985	0.9990	0.9950	1.0141
50-2	1.0319	0.9991	0.9985	0.9990	0.9950	1.0233
50-3	1.0387	0.9991	0.9985	0.9990	0.9950	1.0300

a The product of the correction factors for beam radial nonuniformity and beam axial nonuniformity, *k*_rn_ and *k*_an_, respectively, is unity.

**Table 10:** Correction factors and physical parameters for the ENEA graphite cavity chamber

Saturation loss correction factor^a^	*k*_sat_	1.0028
Radiation scattered by stem correction factor	*k*_st_	1.000
Non-point source effects	*k*_npn_	1.000
Point source nonuniformity	*k*_pn_	1.0001
Wall thickness correction factor	*k*_c_	1.0164
Electron production origin correction factor	*k*_cep_	0.9972
Wall effect correction factor	*k*_w_ = *k*_c_ *k*_cep_	1.0136
Air density correction factor^b^	*k*_Tp_	
Air humidity correction factor [20]^b^	*k*_h_	
Restricted mass stopping power ratio of graphite to air [25]	${\left(L/\rho \right)}_{\text{air}}^{C}$	1.000
Air to carbon mass energy absorbtion coefficient ratio [26, 27]	${\left(\mu_{\text{en}}/\rho \right)}_{C}^{\text{air}}$	0.9985

a At air kerma rate used for the present comparison specified in Table 2.

b The values of correction factors *k*_tp_ and *k*_h_ (temperature, pressure and humidity) were determined according to ambient conditions during measurements.

**Table 11:** Summary of NIST-T1 and NIST-T2 transfer chambers calibration factors at radiation beam codes used for present comparison, as determined at NIST

Transfer chamber		NIST-T1					NIST-T2 Uncertainty	
Beam code	HVL	No. of obs.	Cal. factor^a^	Rel. Uncert.^b^		Cal. factor^a^		
	Cu, mm		10^6^ Gy · C^−1^	*u*_c_	*U*	10^6^ Gy · C^−1^	*u*_c_	*U*
P6	0.1455	18	7.8202	0.04 %	0.09 %	8.4071	0.03 %	0.07 %
P7	0.4835	18	7.9308	0.04 %	0.08 %	8.4923	0.03 %	0.07 %
P8	0.9672	18	8.0308	0.03 %	0.06 %	8.6074	0.03 %	0.06 %
P9	2.4932	18	8.1216	0.04 %	0.08 %	8.7371	0.03 %	0.07 %
^60^Co	14.9	22	8.2123^c^	0.05 %	0.10 %	8.9386^c^	0.04 %	0.09 %

a Combined before and after ENEA comparison, corrected for recombination.

b *u*_c_ is the combined standard uncertainty (i.e., one standard deviation estimate) and $U=k_{u_{c}}$ is the expanded uncertainty with a coverage factor of *k* = 2 (i.e., a 95 % level of confidence estimate).

c Equilibrium shells added.

**Table 12:** Relative standard uncertainties relevant to the air kerma and the exposure determination by the ENEA medium-energy free-air chamber

Source of component of relative standard uncertainty	Air-kerma rate		Exposure rate	
	Type A (%)	Type B (%)^a^	Type A (%)	Type B (%)^a^
*k*_sat_	0.01	0.1	0.01	0.1
*k*_e_		0.15		0.15
*k*_w_		0.06		0.06
*k*_t_		0.1		0.1
*k*		0.05		0.05
*k*_a_	0.02	0.2	0.02	0.2
*k*_P_	0.01	0.03	0.01	0.03
*k*_T_	0.01	0.02	0.01	0.02
*k*_h_		0.05		0.05
Leakage		0.01		0.01
*V*		0.12		0.12
*ρ*		0.02		0.02
*I*	0.05^b^	0.1^c^	0.05^b^	0.1 c
(*W*/*e*) [21]		0.18		Not applied
*g* [21]		0.02		Not applied

Quadratic sum (%)	0.06	0.39	0.06	0.34

Relative combined standard uncertainty, *u*_c_(%)	0.39		0.35	

a The uncertainty of some correction factors that, once determined, are not currently evaluated is considered only of Type B even if a statistical component was included at the time of their experimental determination.

b Standard deviation of the mean of four series of 30 measurements, two with positive and two with negative chamber polarizing voltages, respectively.

c This value includes relative standard uncertainties for voltage (0.06 %), capacitance (0.05 %), time (0.01 %), stray capacitance (0.01 %) and was evaluated by also taking into account the deviations among absolute charge measurements by different measuring systems.

**Table 13:** Relative standard uncertainties associated with the air kerma and exposure rate determinations by the ENEA graphite cavity chamber

Source of component of relative standard uncertainty	Air-kerma rate		Exposure rate	
	Type A	Type B	Type A	Type B
*k*_sat_	0.01	0.1	0.01	0.1
*k*_c_		0.05		0.05
*k*_cep_		0.2		0.2
*k*_st_		0.03		0.03
*k*_rn_		0.1		0.1
*k*_an_		0.1		0.1
*k*_P_	0.01	0.03	0.01	0.03
*k*_T_	0.01	0.02	0.01	0.02
*k*_h_		0.05		0.05
Leakage		0.01		0.01
*V*		0.14		0.14
*ρ*		0.02		0.02
*I*	0.03^b^	0.1^c^	0.03^b^	0.1^c^
${\left(\mu_{\text{en}}/\rho \right)}_{C}^{\text{air}}$		0.1		0.1
${\left(L/\rho \right)}_{air}^{C}$		0.2		0.2
(W/e) [21]		0.18	Not applied	
*g* [21]		0.02	Not applied	

Quadratic sum (%)	0.035	0.44	0.035	0.40

Relative combined standard uncertainty *u*_c_(%)	0.44		0.40	

a The uncertainty of some correction factors that, once determined, are not currently evaluated is considered only of Type B even if a statistical component was included at the time of their experimental determination.

b Standard deviation of the mean of four series of 30 measurements, two with positive and two with negative chamber polarizing voltages, respectively.

c This value includes relative standard uncertainties for voltage (0.06 %), capacitance (0.05 %), time (0.01 %), stray capacitance (0.01 %) and was evaluated by also taking into account the deviations among absolute charge measurements by different measuring systems.

**Table 14:** Relative standard uncertainties relevant to the procedure for calibration of the NIST transfer chambers at ENEA

Source of Component of relative standard uncertainty	Medium energy x-ray				^60^Co gamma radiation			
	Air-kerma		Exposure		Air-kerma		Exposure	
	Type A	Type B	Type A	Type B	Type A	Type B	Type A Type	B
Primary standard	0.06	0.39	0.06	0.34	0.035	0.44	0.035	0.40
Current	0.05^a^	0.1^b^	0.05^a^	0.1^b^	0.05^a^	0.1^b^	0.05^a^	0.1^b^
Recombin. loss		0.1		0.1		0.1		0.1
Distance	0.01		0.01		0.01		0.01	
Pressure		0.02		0.02		0.02		0.02
Temperature		0.03		0.03		0.03		0.03
Humidity		0.05		0.05		0.05		0.05
Leakage		0.02		0.02		0.02		0.02
Radial nonunif.		0.01		0.01		0.01		0.01
Quadratic sum (%)	0.28	0.42	0.28	0.37	0.28	0.47	0.28	0.43
Relative combined standard uncert. *u*_c_ %		0.50	0.46	0.55	0.51			

a Standard deviation of the mean of a series of 30 measurements.

b This value includes relative standard uncertainties for voltage (0.06 %), capacitance (0.05 %), time (0.01 %), stray capacitance (0.01 %) and was evaluated by also taking into account the deviations among absolute charge measurements by different measuring systems.

**Table 15:** Relative standard uncertainties relevant to the air kerma and exposure rate determinations using the NIST medium-energy free-air chamber

Source of component of relative standard uncertainty	Air-kerma rate			Exposure rate	
	Type A (%)	Type B (%)		Type A (%)	Type B (%)
Volume (*S*,*L*)	0.04	0.01		0.04	0.01
Charge (Cap., *V*)	0.03	0.1		0.03	0.1
Timing (*t*)	0.04	0.1		0.04	0.1
Air density (*ρ*)	0.01	0.08		0.01	0.08
Recombination loss (*k*_sat_)		0.1			0.1
Humidity (*k*_h_)		0.1			0.1
Leakage current		0.01			0.01
Radiation background		0.01			0.01
Air attenuation (*k*_a_)		0.07			0.07
Scattered photons (*k*_p_)		0.2			0.07
Electron loss (*k*_e_)		0.1			0.01
Electric field distortion		0.2			0.2
Polarity difference (*k*_±_)	0.03	0.1		0.03	0.1
Aperture penetration		0.04			0.04
Penetration of chamber face		0.01			0.01
(*W*/*e*) [21]		0.18			Not applied
*g* [21]		0.02			Not applied

Quadratic sum (%)	0.07	0.38		0.07	0.33

Relative combined standard uncertainty (%)			0.38	0.34	

**Table 16:** Relative standard uncertainties relevant to the air kerma and exposure rate determinations using the NIST cavities chambers

Source of component of relative standard uncertainty	Air-kerma rate		Exposure rate	
	Type A (%)	Type B (%)	Type A (%)	Type B (%)
Volume (*S*,*L*)	0.06	0.05	0.06	0.05
Charge (Cap., *V*)	0.03	0.1	0.03	0.1
Timing (*t*)	0.04	0.1	0.04	0.1
Air density (*ρ*)	0.01	0.08	0.01	0.08
Recombination loss (*k*_sat_)		0.1		0.1
Humidity (*k*_h_)		0.1		0.1
Leakage current		0.01		0.01
Radiation background		0.01		0.01
Stopping-power ratio		0.25		0.25
Energy-absorption coefficient ratio		0.05		0.05
Stem scatter		0.1		0.1
Mean origin of electrons		0.05		0.05
Effective measurement point		0.05		0.05
Axial nonuniformity		0.02		0.02
Radial nonuniformity		0.01		0.01
(*W*/*e*) [21]		0.18	Not applied	
*g* [21]		0.02	Not applied	

Quadratic sum (%)	0.08	0.40	0.08	0.36

Relative combined standard uncertainty (%)	0.41		0.37	

**Table 17:** Results of the comparison measurements using the NIST transfer chambers NIST-T1 and NIST-T2. *F*_t_ are the transfer chambers calibration factors at reference conditions: *T* = 295. 15 K, *P* = 1013. 25 Pa, and *H* = 50 %. The results are reported as percent deviations, *Δ* (ENEA-NIST), between the calibration factors, for each of the two NIST transfer chambers predicted from measurements made at NIST and those determined at ENEA (see text for details). For each beam code the mean values of the percent deviations are reported

Beam code	NIST-T1			NIST-T2			
	*F*_t_ (10^6^ Gy C^−1^)		*Δ* (%)^a^	*F*_t_ (10^6^ Gy C^−1^)		*Δ* (%)^a^	*Δ*_m_ (%)^b^
	ENEA	NIST	ENEA-NIST	ENEA	NIST	ENEA-NIST	ENEA-NIST
P6	7.772	7.822	− 0.63	8.329	8.408	− 0.93	− 0.78
P7	7.910	7.935	− 0.32	8.451	8.497	− 0.53	− 0.43
P8	8.046	8.036	+ 0.13	8.613	8.614	− 0.01	+ 0.06
P9	8.163	8.122	+ 0.50	8.780	8.738	+ 0.48	+ 0.49
^60^Co^c^	8.215	8.212	+ 0.04	8.94	28.939	+ 0.03	+ 0.03

a *Δ*_=_ {[*F_t_*(ENEA) − *F*_t_(NIST)]/*F*_t_(NIST)} × 100 %.

b *Δ*_m_ = average percent difference

c Equilibrium shells added.

## References

[1] Laitano RF, Pani R, Pellegrini R, Toni MP (1989). Energy distributions and air kerma rates of ISO and BIPM reference filtered x-radiations. ENEA RT/PAS(89).

[2] International Commission on Radiation Units and Measurements (1970). Specification of high activity gamma-ray sources. ICRU Report.

[3] Attix FH (1961). Report NRL-5646.

[4] Laitano RF, Toni MP (1983). The primary exposure standard of ENEA for medium energy X-ray: characteristics and measurement procedures. ENEA Report RT/PROT(83).

[b5-j34lai] 5NBS Handbook 85, Physical Aspects of Irradiation (1964)

[6] Laitano RF, Toni MP (1983). The primary exposure standard for Co-60 gamma radiation: characteristics and measurement procedures. ENEA Report RT/PROT(83).

[7] Wyckoff HO, Attix FH (1957). Design of free-air ionization chambers.

[b8-j34lai] 8International Commission on Radiological Units and Measurements, National Bureau of Standards (U.S.), ICRU Handbook 78 (1959)

[9] Loftus TP, Weaver JT (1974). Standardization of ^60^Co and ^137^Cs Gamma-Ray Beams in Terms of Exposure. J Res Natl Bur Stand (US).

[10] Kara-Michailova E, Lea DE (1940). Recombination in ionization chambers. Proc Camb Phil Soc.

[11] Mie G (1904). Ionization chambers. Ann Phys (Leipzig).

[12] Boutillon M, Niatel MT (1973). A study of a graphite cavity chamber for absolute exposure measurements of ^60^Co gamma rays. Metrologia.

[13] Niatel MT, Loftus TP, Oetzmann W (1975). Comparison of exposure standards for ^60^Co gamma rays. Metrologia.

[14] Bijelaiew AF (1990). An analytic theory of the point-source non-uniformity correction factor for thick-walled ionization chambers in photon beams. Phys Med Biol.

[15] Bielajew AF, Rogers DWO (1992). Implications of new correction factors on primary air kerma standards in Co-60 beams. Phys Med Biol.

[16] Kondo S, Randolph ML (1960). Effect of finite size of ionization chambers on measurements of small photon sources. Radiat Res.

[17] Rogers DWO, Bielajew AF (1990). Wall attenuation and scatter corrections for ion chambers: measurements versus calculations. Phys Med Biol.

[18] Bielajew AF (1990). On the technique of extrapolation to obtain wall correction factors for ion chambers irradiatad by photon beams. Med Phys.

[b19-j34lai] 19International Commission on Radiation Units and Measurements, Radiation Quantities and Units, ICRU Report 33 (1980)

[20] Niatel MT (1969). Rayons X-Etude experimentale de l’influence de la vapeur d’eau sur l’ionization produite dans l’air. C R Acad Sc Paris (Seriès B).

[b21-j34lai] 21BIPM—CCEMRI (Section I), 8 (1985)

[b22-j34lai] 22BIPM, Rapport du groupe de travail sur l’expression des incertitudes au CIPM, BIPM Proc. Verb. Com. Poids et Mesures 49, Annexe A (1981)

[23] International Organization for Standardization (1993). Guide to the expression of uncertainty in measurement.

[24] Taylor BN, Kuyatt CE (1994). Guidelines for evaluating and expressing the uncertainty of NIST measurement results.

[25] Niatel MT, Perrouche-Roux AM, Boutillon M (1985). Two determinations of W. Phys Med Biol.

[26] Hubbell JH (1977). Photon mass attenuation and mass energy-absorption coefficients for H, C, O, Ar, and seven mixtures from 0.1 keV to 20 MeV. Rad Res.

[27] Hubbell JH (1982). Photon mass attenuation and energy-absorption coefficients from 1 keV to 20 MeV. Int J Appl Radiat Isot.

